# Chromatic hyper Zagreb topological descriptors and QSPR analysis of certain skin cancer drugs

**DOI:** 10.3389/fchem.2026.1848388

**Published:** 2026-05-29

**Authors:** R. Pavithra, J. Ravi Sankar

**Affiliations:** Department of Mathematics, School of Advanced Sciences, Vellore Institute of Technology, Vellore, Tamil Nadu, India

**Keywords:** chromatic topological indices, physicochemical properties, QSPR model, regression model, skin cancer drugs

## Abstract

Skin cancer is one of the most common diseases, and studying its drugs is important for effective treatment and drug design. Drugs for melanoma, immunosuppressants, and topical drug administration have a bright future in the investigation of skin cancer drugs with possible anticancer properties. The use of chromatic topological descriptors is still the predominant strategy due to the notable progress being made in the field of drug creation. In this work, the investigation of regression models and chromatic hyper Zagreb topological descriptors for some skin cancer drugs is the main focus of this study. Together with the QSPR models, descriptors provide a numerical representation of a molecule’s chemical characteristics. Proper coloring is applied to the molecular graphs such that no two adjacent vertices share the same color. Regression models are constructed for the calculated index values while the physicochemical properties of skin cancer drugs are investigated. The framework that connects chemical composition to physical attributes is represented by numbers associated with chromatic topological indices. Based on the data collected, an analysis is conducted for a number of noteworthy findings.

## 1 Introduction

The biggest organ in the human body is the skin, which acts as a barrier to protect the body from external dangers, including infections, dangerous UV rays, and chemical exposure. There are three main layers, which are the epidermis, the outermost layer, which is made up of keratinocytes that give structure and melanocytes that produce pigment (Maranke, 2009). The second is the dermis, an intermediate layer that contains sweat glands, connective tissues, blood vessels, nerve endings, and hair follicles, and the last is the hypodermis (subcutaneous tissue), the body’s lowest layer, made up of connective tissues and fat, which aids in insulating and cushioning.

In addition to avoiding dehydration and facilitating sensory awareness, the skin is essential for controlling body temperature. However, cellular damage can result from extended exposure to dangerous UV radiation from the sun or artificial sources and increase the risk of skin cancer (Queen, 2017). Skin cancer is characterized by aberrant skin cell development that results from DNA alterations that cause unchecked cell division. It is one of the most prevalent forms of cancer on the globe and comes in three main varieties: BCC is the most prevalent and least dangerous kind of skin cancer that arises in the epidermis’ basal cells (Dika et al., 2020). The squamous cells in the epidermis give birth to squamous cell carcinoma (SCC), which is more likely to spread than BCC. Originating from melanocytes, melanoma is the most aggressive and lethal kind of skin cancer (Hunter Shain and Bastian, 2016). In the event that melanoma is not identified and treated promptly, it is likely to spread. Moreover, cutaneous lymphoma and merkel cell carcinoma are less frequent types (Becker et al., 2017).

Knowing a drug’s physicochemical, biological, and pharmacological characteristics requires a comprehension of its molecular structure. In graph theory, a graph G=(V,E) is an ordered pair consisting of a non-empty set of vertices V (or nodes/points) and a set of edges E (lines or arcs) that connect pairs of vertices (Zhang and Chartrand, 2006). It represents pairwise relationships between objects, where edges define connections between vertices. In graph theory, a simple graph is an undirected graph that contains no loops and no multiple edges. It is the most basic representation of relationships, where each pair of vertices is connected by at most one edge. Topological indices are numerical invariants derived from molecular graphs, which represent atoms as vertices and bonds as edges that quantify structural features to predict physicochemical properties (Balaban and Ivanciuc, 2000). They offer important information on molecular similarities, activity prediction, and drug design. In chemical graph theory, QSAR is a method that relates the biological activity and molecular structure of a compound whereas QSPR relates a mathematical relationship between the molecular structure of a compound and its physicochemical properties. In chemistry, degree-based topological indices are numerical graph invariants that are used to connect molecule structures with physical and chemical properties such as stability and boiling point without the need for synthesis. Common indices used in QSAR/QSPR investigations are the Randić index, Zagreb indices, and atom-bond connectivity indices, which are calculated by summing functions of vertex degrees $\left(d_{v}\right)$ (Gutman, 2013).

Chemical graph theory is a subfield of mathematical chemistry that applies graph theory to represent and analyze molecular structures, where vertices denote atoms and edges represent chemical bonds (Trinajstic, 2018). Chemical graph theory, drug design, and computational chemistry all make extensive use of topological indices, which offer a mathematical method for examining molecular structures. Colakoglu talks about past research on potential drugs to treat COVID-19 (Çolakoğlu, 2022). Given that it is a costly and intricate phenomenon, this method is most effective when used to forecast discoveries. By using QSPR modeling, Nasir et al.’s blood cancer medication results in demonstrate a substantial relationship between pharmacological properties and TDs (Nasir et al., 2022). Drug design, molecular similarity analysis, and QSAR/QSPR research all make extensive use of distance-based topological indices. Here are some results with topological descriptors in molecular graphs. Two regression methods were examined by Pandeeswari et al. to determine the precision of their forecast by analyzing topological indicators in medications for breast cancer (Pandeeswari and Ravi Sankar, 2025; Pandeeswari and Sankar J, 2025). They offer crucial information on reaction processes, toxicity prediction, boiling temperatures, and molecule stability. Graph energy and topological descriptors of zero divisor graphs was determined by Clement Johnson et al., which associate with commutative rings and provide a strong bridge between spectral graph theory and algebraic structure (Johnson Rayer and Jeyaraj, 2023; Johnson and Sankar, 2023; Clement Johnson Rayer and Ravi Sankar Jeyaraj, 2023). Simran Kour et al. combine machine learning regression approaches and examine a characterization of tricyclic antidepressant drugs and anti-cancer drugs properties by capturing complex relationships between biological activity and molecular descriptors (Kour and Sankar, 2025; Kour and Sankar, 2024).

C. Glory et al. investigate b-chromatic and rainbow chromatic topological indices for various graphs and their derived and central structures, capturing significant relationships between graph coloring techniques and structural properties (Glory and Nanjappa, 2023; Glory et al., 2024). C. Yogalakshmi et al. develop a QSPR graph model using novel coloring-based topological indices to explore physicochemical properties of potential antiviral drugs, capturing complex relationships between molecular structure and drug activity (Yogalakshmi and Balamurugan, 2025). Based on the lower bound for the second hyper-Zagreb index of trees with a given Roman domination number, providing key theoretical insights into graph invariants and structural properties established by Ali et al. (2025)). R. Rajambigai et al. examine the structure–efficiency relationship of access group antibiotics via SK chromatic descriptors, effectively linking molecular topology with antibiotic performance (Rajambigai et al., 2026). In particular, we highlight the fundamental molecule structure that has a greater direct influence on molecular characteristics and biological processes by using hydrogen-depleted graphs, which do not include hydrogen atoms (Gross et al., 2018).

This study aims to develop regression models for predicting QSPR based on physicochemical properties while simultaneously computing chromatic hyper Zagreb topological indices for 10 skin cancer drugs using proper coloring. The reliability and predictive accuracy of these models are assessed by systematically comparing calculated and experimental values. Classical topological indices are widely used to describe molecular structures based on connectivity patterns. However, chromatic Zagreb indices extend these descriptors by including vertex coloring, which provides additional structural information about the molecule. By integrating chemical graphs with molecular descriptor analysis, this work provides a comprehensive topological and physicochemical characterization of the compounds. Moreover, the study exhibits the adaptability of this approach across different classes of skin cancer drugs, highlighting its potential as a powerful tool for drug design and structure–activity relationship studies, and it encourages the creation of new drugs for potential treatment.

## 2 Formulas and methods

**FIGURE 1:**
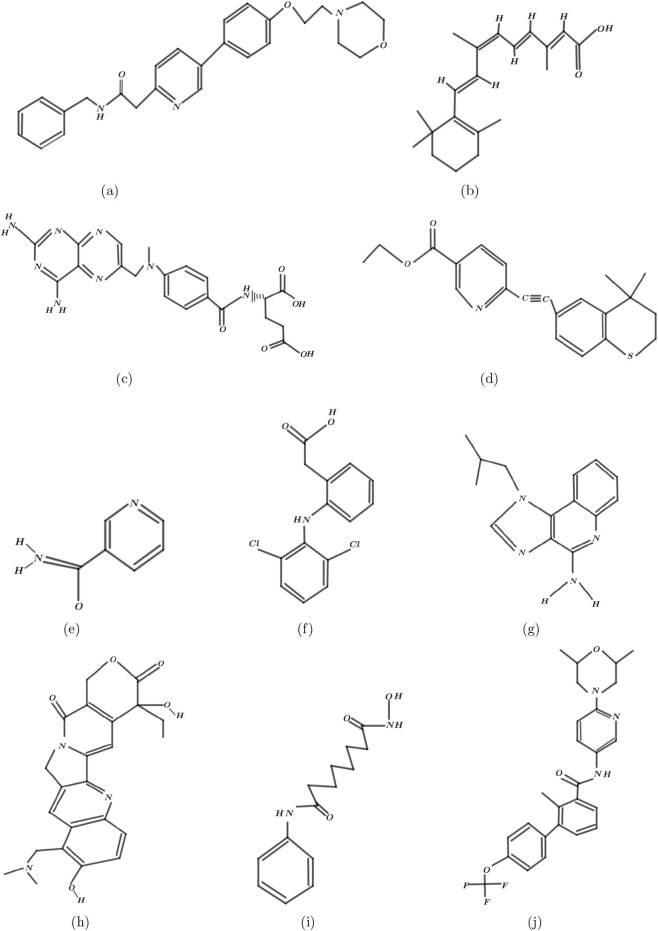
Molecular structures of skin cancer drugs. **(a)** Tirbanibulin. **(b)** Alitretinoin. **(c)** Methotrexate. **(d)** Tazarotene. **(e)** Nicotinamide. **(f)** Diclofenac. **(g)** Imiquimod. **(h)** Topotecan. **(i)** Vorinostat. **(j)** Sonidegib.

**FIGURE 2:**
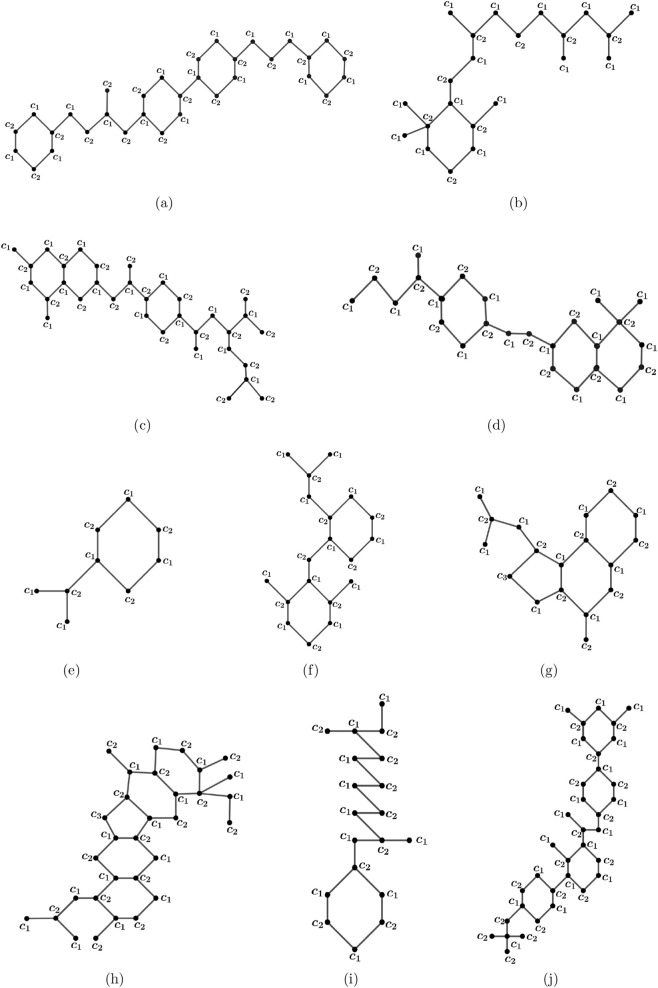
Proper coloring of molecular graphs of skin cancer drugs. **(a)** Tirbanibulin. **(b)** Alitretinoin. **(c)** Methotrexate. **(d)** Tazarotene. **(e)** Nicotinamide. **(f)** Diclofenac. **(g)** Imiquimod. **(h)** Topotecan. **(i)** Vorinostat. **(j)** Sonidegib.

Ten skin cancer drugs were selected for analysis in this study, and the PubChem (National Center for Biotechnology Information NCBI, 2026) and ChemSpider databases (ChemSpider, 2025) were used to gather information on their physicochemical properties. The drugs considered in this study, namely, Tirbanibulin, Alitretinoin, Methotrexate, Tazarotene, Nicotinamide, Diclofenac, Imiquimod, Topotecan, Vorinostat, and Sonidegib, are commonly used in the treatment of skin cancer and related dermatological conditions. Table 1 shows the experimental data for the physical and chemical characteristics of the ten skin cancer drugs, which are shown in Figure 1. As a result, the regression model is a good test to use and examine the data. Figure 2 illustrates the proper coloring of the molecular graphs corresponding to certain skin cancer drugs. This step is essential for the computation of chromatic topological indices, as these indices depend on the assignment of colors to vertices such that no two adjacent vertices share the same color.

**TABLE 1:** Skin cancer drugs with their physicochemical properties.

Drug	Molecular weight (MW)	Molar refractivity (MR)	Polarizability (P)	Complexity (C)	Polar surface area (PSA)	Molar volume (MV)	Surface tension (ST)	log P
Tirbanibulin	431.5	124.2	49.2	540	64	369.0	48.7	1.97
Alitretinoin	300.4	95.5	37.9	567	37	297.2	39.1	6.83
Methotrexate	454.5	119.0	47.2	704	211	295.7	96.5	−0.24
Tazarotene	351.5	101.4	40.2	547	64	287.0	57.5	6.22
Nicotinamide	122.2	33.5	13.3	114	57	99.1	50.2	−0.24
Diclofenac	318.1	76.5	30.3	310	49	206.8	58.0	4.06
Imiquimod	240.3	71.0	28.2	294	57	187.7	48.8	3.46
Topotecan	421.4	112.7	44.7	867	103	281.3	82.9	1.08
Vorinostat	264.3	73.5	29.1	276	78	225.0	50.4	0.86
Sonidegib	485.5	126.7	50.2	691	64	386.8	43.1	5.42

### 2.1 Chromatic modified and chromatic hyper Zagreb indices

The formula was provided in our earlier unpublished research paper (Pavithra, 2026).

Chromatic modified first Zagreb index:

M1ΦmG=∑v∈VG1cv2=∑j=1l1θcj j2 (1)

Chromatic modified second Zagreb index:

M2ΦmG=∑uv∈EG1cucv=∑1≤t,s≤χGt<s1ts ηts (2)

Chromatic first hyper Zagreb index:

$$HM_{1}^{\Phi }\left(G\right)=\underset{uv\in E\left(G\right)}{\sum }{\left[c\left(u\right)+c\left(v\right)\right]}^{2}=\overset{t<s}{\underset{1\leq t,s\leq \chi \left(G\right)}{\sum }}\eta_{ts}{\left[t+s\right]}^{2}$$ (3)

Chromatic second hyper Zagreb index:

$$HM_{2}^{\Phi }\left(G\right)=\underset{uv\in E\left(G\right)}{\sum }{\left[c\left(u\right)c\left(v\right)\right]}^{2}=\overset{t<s}{\underset{1\leq t,s\leq \chi \left(G\right)}{\sum }}\eta_{ts}{\left[ts\right]}^{2}$$ (4)

where $\theta \left(c_{j}\right)$ is the strength or cardinality of a color class $c_{j}$ ; 1≤j≤l . Also, it $\eta_{ts}$ denotes the number of edges with end points t and s, where t and s are colors in the set of colors $C=\left\{c_{1},c_{2},..,c_{l}\right\}$ . By computing the values of $\theta \left(c_{j}\right)$ and $\eta_{ts}$ and replacing them in the preceding formulas, the computed values of the chromatic modified and chromatic hyper Zagreb indices of the ten skin cancer drugs are displayed in Table 2. By using Equations 1–4, the chromatic modified and chromatic hyper Zagreb indices of the molecular graph are computed and displayed in Table 2.

**TABLE 2:** Chromatic topological indices for skin cancer drugs.

Drug	M1Φm(G)	M1Φm(G)	$HM_{1}^{\Phi }\left(G\right)$	$HM_{2}^{\Phi }\left(G\right)$
Tirbanibulin	0.078125	0.01428	315	140
Alitretinoin	0.10267	0.0227	198	88
Methotrexate	0.0772	0.01428	315	140
Tazarotene	0.0941	0.018	243	108
Nicotinamide	0.2625	0.0556	81	36
Diclofenac	0.12215	0.025	180	80
Imiquimod	0.25347	0.52778	203	117
Topotecan	0.1944	0.3489	345	182
Vorinostat	0.12778	0.0263	171	76
Sonidegib	0.07026	0.01315	342	152

## 3 Regression model

This study makes use of the 8 physicochemical characteristics listed in Table 1. Correlations between chromatic topological indices and several physicochemical properties of skin cancer drugs are found using the formula. The model of linear regression used in this work is

$$P=A+B\left(TI^{\phi }\right)$$ (5)

Here, P represents the physicochemical properties of the drugs, and the chromatic topological index values of the corresponding drugs are represented by $TI^{\phi }$ , A for a constant term and B for the regression coefficient in the formula above. By analyzing both the physicochemical properties and chromatic topological index values of the ten skin cancer drugs, the parameters A and B are computed using SPSS software and Microsoft Excel.

In this analysis, the chromatic topological indices of the molecular graphs of the skin cancer drugs are treated as independent variables, whereas the physicochemical properties are treated as dependent variables. Using Equation 5, the resulting regression models for the chromatic topological indices are obtained, as represented in Table 3. Table 4 illustrates the correlation coefficients of the chromatic modified and chromatic hyper Zagreb indices against the eight physicochemical properties. Strong correlations are highlighted in bold.

**TABLE 3:** Regression models of physicochemical properties with chromatic topological indices.

Properties	M1Φm(G)	M2Φm(G)	$HM_{1}^{\Phi }\left(G\right)$	$HM_{2}^{\Phi }\left(G\right)$
MW	497.35−1145.54[M1Φm(G)]	351.21−114.90[M2Φm(G)]	$44.79+1.229\left[HM_{1}^{\Phi }\left(G\right)\right]$	$87.77+2.244\left[HM_{2}^{\Phi }\left(G\right)\right]$
MR	135.87−307.19[M1Φm(G)]	96.29−27.11[M2Φm(G)]	$16.47+0.3214\left[HM_{1}^{\Phi }\left(G\right)\right]$	$27.53+0.588\left[HM_{2}^{\Phi }\left(G\right)\right]$
P	53.83−121.53[M1Φm(G)]	38.164−10.63[M2Φm(G)]	$6.54+0.127\left[HM_{1}^{\Phi }\left(G\right)\right]$	$10.918+0.233\left[HM_{2}^{\Phi }\left(G\right)\right]$
C	718.40−1644.75[M1Φm(G)]	489.11+17.70[M2Φm(G)]	$-92.73+2.439\left[HM_{1}^{\Phi }\left(G\right)\right]$	$-47.84+4.815\left[HM_{2}^{\Phi }\left(G\right)\right]$
PSA	99.99−156.20[M1Φm(G)]	79.98−14.86[M2Φm(G)]	$17.55+0.254\left[HM_{1}^{\Phi }\left(G\right)\right]$	$25.177+0.475\left[HM_{2}^{\Phi }\left(G\right)\right]$
MV	397.03−965.37[M1Φm(G)]	278.37−138.99[M2Φm(G)]	$62.24+0.841\left[HM_{1}^{\Phi }\left(G\right)\right]$	$100.54+1.456\left[HM_{2}^{\Phi }\left(G\right)\right]$
ST	59.37−13.38[M1Φm(G)]	56.24+11.94[M2Φm(G)]	$36.23+0.0889\left[HM_{1}^{\Phi }\left(G\right)\right]$	$35.91+0.193\left[HM_{2}^{\Phi }\left(G\right)\right]$
log⁡P	4.794−13.39[M1Φm(G)]	3.109−1.57[M2Φm(G)]	$2.405+0.002\left[HM_{1}^{\Phi }\left(G\right)\right]$	$2.744+0.0017\left[HM_{2}^{\Phi }\left(G\right)\right]$

**TABLE 4:** Correlation coefficient of physicochemical properties.

Index	Molecular weight (MW)	Molar refractivity (MR)	Polarizability (P)	Complexity (C)	Polar surface area (PSA)	Molar volume (MV)	Surface tension (ST)	log P
M1Φm(G)	**0.737**	**0.751**	**0.750**	0.507	0.227	**0.813**	0.053	0.368
M2Φm(G)	0.183	0.164	0.163	0.013	0.053	0.290	0.118	0.107
$HM_{1}^{\Phi }\left(G\right)$	**0.957**	**0.951**	**0.951**	**0.911**	0.448	**0.857**	0.430	0.074
$HM_{2}^{\Phi }\left(G\right)$	**0.859**	**0.856**	**0.857**	**0.883**	0.412	**0.729**	0.459	0.028

Bold value indicate strong correlation coefficients (r > 0.7).

**FIGURE 3:**
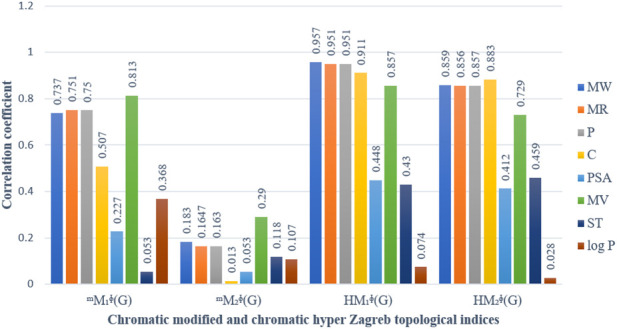
Graphical representation of the correlation coefficient and physicochemical properties of the drugs.

Figure 3 displays the graph of the correlation coefficient of chromatic modified and chromatic hyper Zagreb indices and all the physicochemical properties, including molecular weight, molar refractivity, polarizability, complexity, polar surface area, molar volume, surface tension and log P. To evaluate the relationship between physicochemical properties and chromatic topological indices, correlation coefficients were created.

## 4 Standard estimate error and statistical metrics evaluation

The statistical parameters are combined for all physicochemical properties and chromatic topological indices in Tables (5–8) to assist us in understanding how they relate to each other. A number of statistical characteristics are observed, including the significant value p, Fisher’s statistic, the percentage of dependent variables $R^{2}$ , the slope b, the constant term A, the sample size N, and the importance of the association for each of the chromatic topological indices. From an interpretive standpoint, a p-value less than 0.05 is considered statistically significant, whereas a p-value of more than 0.05 is seen as not being statistically significant.

**TABLE 5:** Statistical parameters of the linear QSPR model using M1Φm(G) .

Properties	N	A	B	$R^{2}$	F	p	Indicator
MW	10	497.35	−1145.54	0.543	9.512	0.015	Significant
MR	10	135.87	−307.19	0.5646	10.37	0.012	Significant
P	10	53.83	−121.53	0.563	10.31	0.012	Significant
C	10	718.40	−1644.75	0.257	2.77	0.134	Insignificant
PS	10	99.99	−156.20	0.051	0.437	0.526	Insignificant
MV	10	397.03	−965.37	0.661	15.60	0.004	Significant
ST	10	59.37	−13.38	0.0028	0.022	0.883	Insignificant
log P	10	4.794	−13.39	0.136	1.260	0.294	Insignificant

**TABLE 6:** Statistical parameters of the linear QSPR model using M2Φm(G) .

Properties	N	A	B	$R^{2}$	F	p	Indicator
MW	10	351.21	−114.90	0.033	0.279	0.611	Insignificant
MR	10	96.29	−27.11	0.0271	0.223	0.6493	Insignificant
P	10	38.164	−10.63	0.026	0.218	0.652	Insignificant
C	10	489.11	17.70	0.0001	0.0014	0.97	Insignificant
PS	10	79.98	−14.86	0.0028	0.0232	0.882	Insignificant
MV	10	278.37	−138.99	0.084	0.738	0.415	Insignificant
ST	10	56.24	11.94	0.014	0.114	0.7443	Insignificant
log P	10	3.109	−1.57	0.0115	0.093	0.767	Insignificant

**TABLE 7:** Statistical parameters of the linear QSPR model using $HM_{1}^{\Phi }\left(G\right)$ .

Properties	N	A	B	$R^{2}$	F	p	Indicator
MW	10	44.79	1.229	0.916	87.54	0.00001	Significant
MR	10	16.47	0.3214	0.9057	76.86	0.00002	Significant
P	10	6.54	0.127	0.906	77.203	0.00002	Significant
C	10	−92.73	2.439	0.829	39.04	0.0002	Significant
PS	10	17.55	0.254	0.201	2.016	0.193	Insignificant
MV	10	62.24	0.841	0.735	22.22	0.0015	Significant
ST	10	36.23	0.0889	0.185	1.817	0.214	Insignificant
log P	10	2.405	0.002	0.0055	0.044	0.837	Insignificant

**TABLE 8:** Statistical parameters of the linear QSPR model using $HM_{2}^{\Phi }\left(G\right)$ .

Properties	N	A	B	$R^{2}$	F	p	Indicator
MW	10	87.77	2.2244	0.738	22.55	0.001	Significant
MR	10	27.53	0.588	0.733	22.03	0.001	Significant
P	10	10.918	0.233	0.734	22.14	0.001	Significant
C	10	−47.84	4.815	0.781	28.59	0.0006	Significant
PS	10	25.177	0.475	0.170	1.640	0.236	Insignificant
MV	10	100.54	1.456	0.532	9.12	0.016	Significant
ST	10	35.91	0.193	0.210	2.135	0.182	Insignificant
log P	10	2.744	0.0017	0.0008	0.006	0.936	Insignificant

These measures not only enable comparisons but also provide accurate computations. The physicochemical properties of the skin cancer drugs are listed with the standard estimation error in Table 9. The accuracy of the QSPR model predictions are facilitated by the computation of standard estimation error. The comparison between the calculated and observed values of physicochemical properties, based on the regression models of chromatic modified and chromatic hyper Zagreb indices, are presented in Tables 10–17. The correlation determination provides more information about the relationship between variables by describing the percentage of the association.

**TABLE 9:** Standard estimate error for physicochemical properties of the drugs.

Drug	Molecular Weight (MW)	Molar Refractivity (MR)	Polarizability (P)	Complexity (C)	Polar surface Area (PSA)	Molar Volume (MV)	Surface Tension (ST)	log P
M1Φm(G)	80.87	20.76	8.242	214.97	51.41	53.21	19.23	2.59
M2Φm(G)	117.62	31.04	12.30	249.47	52.72	87.45	19.12	2.779
$HM_{1}^{\Phi }\left(G\right)$	34.62	9.66	3.82	102.88	47.18	47.02	17.39	2.787
$HM_{2}^{\Phi }\left(G\right)$	61.23	16.24	6.423	116.65	48.09	62.48	17.11	2.794

**TABLE 10:** Comparison of observed and predicted values of molecular weight based on regression models.

Drug	Molecular weight	M1Φm(G)	M2Φm(G)	$HM_{1}^{\Phi }\left(G\right)$	$HM_{2}^{\Phi }\left(G\right)$
Tirbanibulin	431.5	407.855	349.569	431.925	401.930
Alitretinoin	300.4	379.712	348.602	288.132	285.242
Methotrexate	454.5	408.915	349.569	431.925	401.930
Tazarotene	351.5	389.546	349.142	343.437	330.122
Nicotinamide	122.2	196.395	344.815	144.339	168.554
Diclofenac	318.1	357.393	348.338	266.010	267.290
Imiquimod	240.3	206.997	290.571	294.277	350.318
Topotecan	421.4	274.609	311.125	468.795	495.778
Vorinostat	264.3	351.939	348.189	254.949	258.314
Sonidegib	485.5	416.875	349.700	465.208	428.658

**TABLE 11:** Comparison of observed and predicted values of molar refractivity based on regression models.

Drug	Molar refractivity	M1Φm(G)	M2Φm(G)	$HM_{1}^{\Phi }\left(G\right)$	$HM_{2}^{\Phi }\left(G\right)$
Tirbanibulin	124.2	111.871	95.903	117.711	109.850
Alitretinoin	95.5	104.328	95.675	80.107	79.274
Methotrexate	119.0	112.155	95.903	117.711	109.850
Tazarotene	101.4	106.965	95.802	94.580	91.034
Nicotinamide	33.5	55.227	94.783	42.503	48.698
Diclofenac	76.5	98.339	95.612	74.322	74.570
Imiquimod	71.0	57.995	81.984	81.714	96.326
Topotecan	112.7	76.148	86.831	127.353	134.546
Vorinostat	73.5	96.623	95.577	71.429	72.218
Sonidegib	126.7	114.289	95.934	126.389	116.906

**TABLE 12:** Comparison of observed and predicted values of polarizability based on regression models.

Drug	Polarizability	M1Φm(G)	M2Φm(G)	$HM_{1}^{\Phi }\left(G\right)$	$HM_{2}^{\Phi }\left(G\right)$
Tirbanibulin	49.2	44.335	38.012	46.545	43.538
Alitretinoin	37.9	41.351	37.923	31.686	31.422
Methotrexate	47.2	44.448	38.012	46.545	43.538
Tazarotene	40.2	42.396	37.973	37.401	36.082
Nicotinamide	13.3	21.930	37.573	16.827	19.306
Diclofenac	30.3	38.983	37.898	29.400	29.558
Imiquimod	28.2	23.025	32.553	32.321	38.179
Topotecan	44.7	30.206	34.455	50.355	53.324
Vorinostat	29.1	38.301	37.884	28.257	28.626
Sonidegib	50.2	45.291	38.024	49.974	46.334

**TABLE 13:** Comparison of observed and predicted values of complexity bases on regression models.

Drug	Complexity	M1Φm(G)	M2Φm(G)	$HM_{1}^{\Phi }\left(G\right)$	$HM_{2}^{\Phi }\left(G\right)$
Tirbanibulin	540	589.888	489.363	675.555	626.260
Alitretinoin	567	549.522	489.512	390.192	375.880
Methotrexate	704	591.408	489.363	675.555	626.260
Tazarotene	547	563.616	489.429	499.947	472.180
Nicotinamide	114	286.741	490.094	104.829	125.500
Diclofenac	310	517.467	489.553	346.290	337.360
Imiquimod	294	301.578	498.452	402.387	515.515
Topotecan	867	398.653	495.285	748.725	828.490
Vorinostat	276	508.214	489.575	324.339	317.100
Sonidegib	691	602.822	489.343	741.308	684.040

**TABLE 14:** Comparison of observed and predicted values of polar surface area based on regression models.

Drug	Polar surface area	M1Φm(G)	M2Φm(G)	$HM_{1}^{\Phi }\left(G\right)$	$HM_{2}^{\Phi }\left(G\right)$
Tirbanibulin	64	87.787	79.768	97.560	91.677
Alitretinoin	37	83.950	79.643	67.842	66.977
Methotrexate	211	87.930	79.768	97.560	91.677
Tazarotene	64	85.292	79.713	79.272	76.477
Nicotinamide	57	58.978	79.154	38.124	42.277
Diclofenac	49	80.908	79.609	63.270	63.177
Imiquimod	57	60.393	72.135	69.112	80.752
Topotecan	103	69.628	74.795	105.180	111.627
Vorinostat	78	80.031	79.589	61.984	61.277
Sonidegib	64	89.014	79.785	104.418	97.377

**TABLE 15:** Comparison of observed and predicted values of molar volume based on regression models.

Drug	Molar volume	M1Φm(G)	M2Φm(G)	$HM_{1}^{\Phi }\left(G\right)$	$HM_{2}^{\Phi }\left(G\right)$
Tirbanibulin	369.0	321.611	276.385	327.155	304.380
Alitretinoin	297.2	297.912	275.215	228.758	228.668
Methotrexate	295.7	322.502	276.385	327.155	304.380
Tazarotene	287.0	306.176	275.868	266.603	257.788
Nicotinamide	99.1	143.606	270.642	130.361	152.956
Diclofenac	206.8	279.101	274.895	213.620	217.020
Imiquimod	187.7	152.379	205.016	232.963	270.892
Topotecan	281.3	209.379	229.879	352.385	365.532
Vorinostat	225.0	273.674	274.714	206.051	211.196
Sonidegib	386.8	329.192	276.542	349.962	321.852

**TABLE 16:** Comparison of observed and predicted values of surface tension based on regression models.

Drug	Surface tension	M1Φm(G)	M2Φm(G)	$HM_{1}^{\Phi }\left(G\right)$	$HM_{2}^{\Phi }\left(G\right)$
Tirbanibulin	48.7	58.325	56.410	64.234	62.93
Alitretinoin	39.1	57.996	56.511	53.832	52.89
Methotrexate	96.5	58.337	56.410	64.234	62.93
Tazarotene	57.5	58.111	56.455	57.833	56.75
Nicotinamide	50.2	55.858	56.904	43.431	42.85
Diclofenac	58.0	57.736	56.538	52.232	51.35
Imiquimod	48.8	55.978	62.543	54.277	58.49
Topotecan	82.9	56.769	60.406	66.901	71.03
Vorinostat	50.4	57.660	56.554	51.432	50.57
Sonidegib	43.1	58.430	56.397	66.634	65.24

**TABLE 17:** Comparison of observed and predicted values of log⁡P based on regression models.

Drug	log P	M1Φm(G)	M2Φm(G)	$HM_{1}^{\Phi }\left(G\right)$	$HM_{2}^{\Phi }\left(G\right)$
Tirbanibulin	1.97	3.748	3.087	3.035	2.982
Alitretinoin	6.83	3.419	3.073	2.801	2.894
Methotrexate	−0.24	3.760	3.087	3.035	2.982
Tazarotene	6.22	3.534	3.081	2.891	2.928
Nicotinamide	−0.24	1.279	3.022	2.567	2.805
Diclofenac	4.06	3.158	3.070	2.765	2.880
Imiquimod	3.46	1.400	2.280	2.811	2.943
Topotecan	1.08	2.192	2.561	3.095	3.053
Vorinostat	0.86	3.083	3.068	2.747	2.873
Sonidegib	5.42	3.854	3.088	3.089	3.002

## 5 Discussion

In this work, we studied the relationship between 8 physicochemical characteristics of ten skin cancer drugs with the four chromatic Zagreb topological indices. Table 4 represents the correlation coefficients between the four chromatic Zagreb topological indices and the eight physicochemical properties, with particular attention paid to the substantial association (r>0.7) that was observed. After looking at everything, the properties showed the moderate to strong correlation with the chromatic modified and chromatic hyper Zagreb indices, with molecular weight (r = 0.957), molar refractivity (r = 0.951), polarizability (r = 0.951), complexity (r = 0.911), molar volume (r = 0.857), which indicates that these chemical characteristics may be well predicted by these chromatic topological indices. These physicochemical properties show negligible because all of the chromatic topological indices exhibit a weak association (r<0.2) with the polar surface area, surface tension and log P.

Tables 5–8 presents the QSPR analysis utilizing statistical features such $R^{2}$ , F-statistic, p-value, and regression coefficients A and B. Notably, molecular weight has $R^{2}=0.916$ , F=87.54 , p=0.00001 , polarizability has $R^{2}=0.906$ , F=77.203 , p=0.00002 , molar refractivity has $R^{2}=0.9057$ , F=76.86 , p=0.00002 , with $HM_{1}^{\Phi }\left(G\right)$ index. Remaining some physical properties shows the moderate dependency on chromatic topological indices such as complexity has $R^{2}=0.829$ , F=39.04 , P=0.0002 and molar volume has $R^{2}=0.735$ , F=22.22 , p=0.0015 with $HM_{1}^{\Phi }\left(G\right)$ index. The polar surface area, surface tension and log P show weaker correlations compared to other properties. This may be due to the fact that these properties depend on complex molecular interactions and environmental factors that are not fully captured by the selected chromatic topological descriptors. Table 6 shows the QSPR model based on the M2Φm(G) index shows insignificant relationships with all the considered physicochemical properties, indicating that this index has limited applicability for predictive modeling in the present study. While examining the correlation determination for physical properties under consideration, we have that $HM_{1}^{\Phi }\left(G\right)$ index gives the moderate to strong correlation determination for molecular weight $\left(R^{2}=0.916\right)$ , molar refractivity $\left(R^{2}=0.9057\right)$ and polarizability $\left(R^{2}=0.906\right)$ , then complexity $\left(R^{2}=0.829\right)$ , molar volume $\left(R^{2}=0.735\right)$ . We can also see high percentage correlation between chromatic modified and chromatic hyper Zagreb index and physical properties of drugs. $HM_{1}^{\Phi }\left(G\right)$ index has positive and high percentage of correlation for molecular weight $\left(R^{2}=0.916\right)$ , molar refractivity $\left(R^{2}=0.9057\right)$ and polarizability $\left(R^{2}=0.906\right)$ .

According to this analysis, the values of the chromatic modified and chromatic hyper Zagreb indices are essential for comprehending the properties that are predicted, such as molecular weight, that helps in comprehending the intermolecular forces that aid in the design of models with particular dielectric properties. Drug design can also benefit from the use of the chromatic modified and chromatic hyper Zagreb index values for estimating molecular weight, molar refractivity, and polarizability, for which the predicted error is quite large.

## 6 Limitations and future directions

This research demonstrates significant correlations between chromatic topological indices and selected physicochemical properties; certain limitations should be acknowledged. First, the analysis is based on a relatively small dataset of ten skin cancer drugs, which may limit the generalizability of the results. Second, the study primarily employs linear regression models, which may not fully capture complex non-linear relationships between molecular descriptors and physicochemical properties. Furthermore, some properties such as non-linear models or hybrid modeling may need to be investigated for greater accuracy to predict polar surface area, surface tension, and log P. The sample size considered in this study is relatively small, which may limit the general applicability of the developed QSPR models. A larger dataset could provide more reliable and generalized results.

Future research may focus on incorporating additional statistical parameters such as Root Mean Square Error (RMSE) and Average Absolute Relative Deviation (AARD) to provide a more detailed evaluation of the QSPR models. Further studies can also explore advanced regression techniques such as quadratic and cubic models and incorporate machine learning approaches to improve predictive accuracy. In order to enhance prediction and guide future research, the study therefore demonstrates the necessity of adjusting QSPR models to characterize physicochemical properties. These directions can significantly contribute to the development of more accurate and efficient computational models for drug design and analysis.

## 7 Conclusion

To comprehend the structural characteristics of skin cancer drugs, four chromatic Zagreb topological indices have been used. The physicochemical properties of some drugs have also been subjected to QSPR investigation. Certain chromatic topological indices are shown to be effective in predicting characteristics like polarizability, molecular weight, and molar refractivity. Thus, the work demonstrates that molecular structure is crucial in controlling the properties of specific drugs, suggesting that chromatic Zagreb topological indices are required to predict these characteristics. The estimated value produced from this will enable the pharmaceutical industry to develop new drugs that will surely be helpful in obtaining preventative measures for the aforementioned illness. For new exposures to different illnesses, they offer methods for estimating characteristics. Entropy, critical pressure, boiling point, acentric factor, enthalpy, and other characteristics and processes may all be determined and predicted with its help. Additionally, our findings may help in the creation of new drugs to treat skin cancer.

## Data Availability

The original contributions presented in the study are included in the article/supplementary material, further inquiries can be directed to the corresponding author.
